# Poorly differentiated adenocarcinoma with signet ring cells of the gallbladder producing a high level of alpha-fetoprotein: A case report

**DOI:** 10.1016/j.ijscr.2022.107780

**Published:** 2022-11-17

**Authors:** Junya Mita, Kazuhiro Tada, Yusuke Kuboyama, Kentaro Iwaki, Shun Nakamura, Kengo Fukuzawa

**Affiliations:** a Department of Surgery, Oita Red Cross Hospital, 3-2-37 Chiyomachi, Oita-shi, Oita, Japan; b Department of Pathology, Oita Red Cross Hospital, 3-2-37 Chiyomachi, Oita-shi, Oita, Japan

**Keywords:** AFP, alpha-fetoprotein, HCC, hepatocellular carcinoma, CE-CT, contrast enhanced computed tomography, CA19–9, carbohydrate antigen 19-9, CEA, carcinoembryonic antigen, PIVKA-II, protein induced by vitamin K absence-II, ICG-R15, indocyanine green retention rate at 15 min, SALL 4, Sal-like protein 4, Gallbladder carcinoma, AFP, Cholecystectomy

## Abstract

**Introduction:**

Alpha-fetoprotein (AFP) can become elevated in hepatocellular carcinoma (HCC), yolk sac tumors and other malignant tumors of various organs. Herein, we present a case of AFP-producing gallbladder carcinoma with signet ring cells successfully treated with laparoscopic whole-layer cholecystectomy.

**Presentation of case:**

A 69-year-old woman was found to have increased levels of the tumor marker AFP (16.1 to 1474 ng/mL), and an irregularly shaped mass 22 mm in size in the gallbladder at 5 months follow-up after transcatheter arterial chemoembolization and radiofrequency ablation for HCC in segment 3 of the liver. As no additional metastases were detected, we diagnosed the patient with either AFP-producing gallbladder carcinoma (cT2aN0M0, cStage IIa, UICC 8th) or gallbladder metastasis from HCC. Laparoscopic whole-layer cholecystectomy was performed, and histological examination revealed AFP positive poorly differentiated adenocarcinoma with signet ring cells (pT2bN0cM0, pStage IIb, UICC 8th). AFP levels were remarkably decreased after operation (15 ng/mL), and no residual tumors or distant metastases were observed on contrast enhanced computed tomography (CE-CT), indicating that the tumor was an AFP-producing gallbladder carcinoma rather than metastasis of HCC.

**Discussion:**

Due to the similar developmental origin of the liver and gallbladder, gallbladder carcinoma could produce AFP in some cases. Considering that AFP is predominantly synthesized during embryogenesis, the status of cellular differentiation would be associated with the ability to synthesize AFP.

**Conclusion:**

When no lesions except for in the gallbladder can account for elevated AFP levels, clinicians should consider AFP-producing gallbladder carcinoma.

## Highlights

• AFP-producing gallbladder carcinoma with signet ring cells is extremely rare.
• The status of cellular differentiation would be associated with the ability to synthesize AFP.
• When no lesions except for in the gallbladder can account for elevated AFP levels, clinicians should consider AFP-producing gallbladder carcinoma.

## 1 Introduction

Although elevated serum levels of carbohydrate antigen 19-9 (CA19-9) and carcinoembryonic antigen (CEA) are often seen in gallbladder carcinoma, increased serum alpha-fetoprotein (AFP) levels are only detected in rare cases. Herein, we present a case of AFP-producing gallbladder carcinoma with signet ring cells successfully treated with laparoscopic whole-layer cholecystectomy.

## 2 Presentation of case

**Fig. 1:**
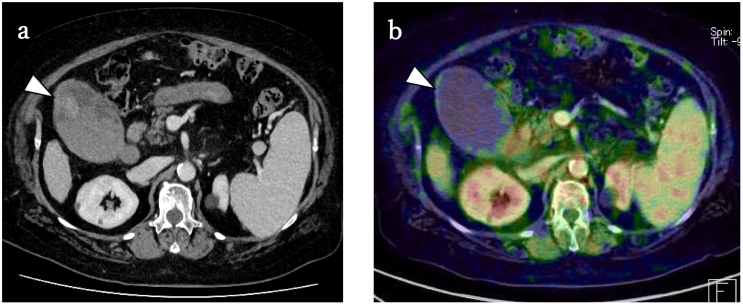
Contrast-enhanced computed tomography (CE-CT) at a follow-up visit 3 months after transcatheter arterial chemoembolization and radiofrequency ablation. (A) Abdominal CE-CT showed an irregularly shaped mass 22 mm in size at the fundus of the gallbladder (arrowhead). (B) Positron emission tomography-CT showed no accumulation in the gallbladder (arrowhead).

**Fig. 2:**
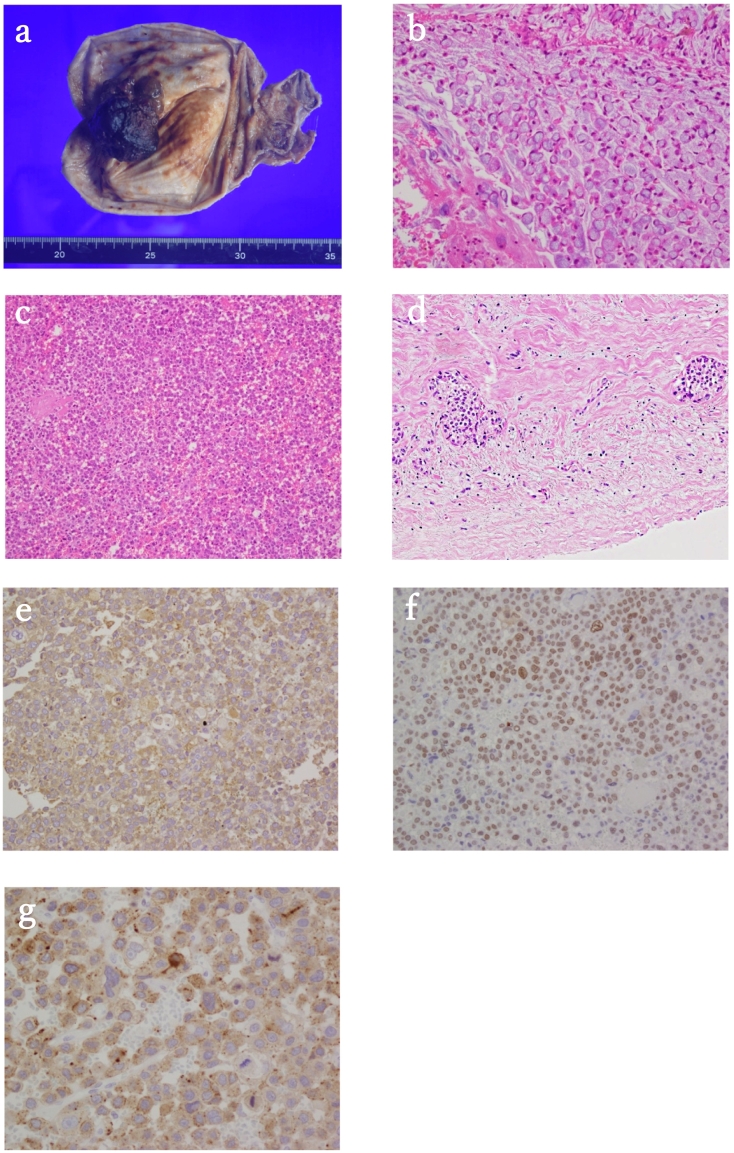
Pathological analysis of the gallbladder carcinoma. (A) Macroscopic image of the resected gallbladder carcinoma, which was a firm black polypoidal tumor measuring 20 × 20 mm. (B-F) Microscopic images of the tumor. (B) Signet ring cells (hematoxylin and eosin staining, original magnification: 400×). (C) Poorly differentiated carcinoma (hematoxylin and eosin staining, original magnification: 200×). (D) The tumor invaded the perimuscular connective tissue on the side of the liver but not spread to the liver (pT2bN0cM0, pStage IIb, UICC 8th) (hematoxylin and eosin staining, original magnification: 200×). (E) Alpha-fetoprotein (AFP) immunohistochemistry (original magnification: 400×). (F) Sal-like protein 4 (SALL 4) immunohistochemistry (original magnification: 400×). (G) Glypican 3 immunohistochemistry (original magnification: 400×).

A 69-year-old woman with non-alcoholic steatohepatitis-related cirrhosis was found to have hepatocellular carcinoma (HCC) in segment 3 of the liver in a follow-up visit 4 years after an initial diagnosis of cirrhosis. The tumor markers CA19-9, AFP, and protein induced by vitamin K absence-II (PIVKA-II) were 35.0 ng/mL, 16.1 U/mL, and 45.1 U/mL, respectively. Two months later, she received transcatheter arterial chemoembolization and radiofrequency ablation. A contrast-enhanced computed tomography (CE-CT) scan 3 months after the intervention showed an accumulation of lipiodol within the tumor and surrounding low-density zone, indicating ablation-induced coagulation necrosis of the liver; no remaining viable tumors were found in the liver. However, an irregularly shaped mass 22 mm in size was found at the fundus of the gallbladder (Fig. 1A), which showed no accumulation of [18F]-fluorodeoxyglucose on positron emission tomography-CT (Fig. 2B).

Through CE-CT, the tumor was thought to have invaded the perimuscular connective tissue on the peritoneal side, without involvement of the serosa (visceral peritoneum). Levels of the tumor marker AFP were significantly increased at 5 months follow-up (16.1 to 1474 ng/mL), and positron emission tomography-CT detected no additional metastases. Therefore, the patient was diagnosed with either AFP-producing gallbladder carcinoma (cT2aN0M0, cStage IIa) or gallbladder metastasis from HCC. In addition to the condition of the liver cirrhosis (ICG-R15 28 %), the patient had type 2 diabetes mellitus with severe obesity (BMI ≥ 35). Considering multiple comorbidities, we performed laparoscopic whole-layer cholecystectomy to minimize postoperative complications. The operation time was 117 min, and the blood loss was 150 mL. Gross inspection of the specimen showed a firm black polypoidal tumor measuring 20 × 20 mm, with necrosis (Fig. 2A). Microscopic examination revealed poorly differentiated adenocarcinoma with signet ring cells (Fig. 2B, C). The tumor invaded the perimuscular connective tissue on the side of the liver but not spread to the liver (pT2bN0cM0, pStage IIb, UICC 8th) (Fig. 2D). Immunohistochemical detection of AFP, Sal-like protein 4 (SALL 4), and Glypican 3 were positive (Fig. 2E-G). The postoperative course was uneventful, and the patient was discharged 12 days following successful surgery. Laboratory tests performed 14 days after discharge showed remarkably decreased AFP levels (15 ng/mL), and no residual tumors or distant metastases were observed on CE-CT, indicating the tumor was AFP-producing gallbladder carcinoma rather than metastasis of HCC. The patient has a good quality of life following this successful treatment.

## 3 Discussion

Gallbladder carcinoma is the fifth most common gastrointestinal malignancy and remains a lethal disease with a poor prognosis [1]. Adenocarcinoma is the most common histological subtype of primary gallbladder carcinoma, representing approximately 80 % to 95 % of all cases. Signet ring cell carcinoma accounts for only 0.5 % to 2 % of all gallbladder cancers [2], [3]. Signet ring cell carcinoma often occurs in organs of the digestive system such as the stomach (86.8 %), colon (3.6 %), and gallbladder (2.5 %) [4]. The biological features of and treatment strategies for signet ring cell carcinoma of the gallbladder have not been well established due to the limited number of cases and studies. Generally, signet ring cell carcinomas have a worse prognosis than adenocarcinomas among gastric and colorectal cancers [5], [6]. These findings suggest that signet ring cell carcinoma of the gallbladder can also be associated with a poor prognosis. Wang S et al. reported that the 5-year overall survival rate of patients with signet ring cell carcinoma was 8.0 %, compared with 14.9 % in patients with non-signet ring cell carcinoma of the gallbladder [7].

In terms of tumor markers, elevated serum CA19-9 and CEA levels are often seen in gallbladder carcinoma [8], [9]. Wang Y et al. found that CA19-9 (but not CEA) levels increased gradually with the clinical progression of gallbladder carcinoma. The sensitivity and specificity of CA19-9 for the diagnosis of gallbladder carcinoma were higher than those of other tumor markers (sensitivity: 71.7 %, specificity: 96.1 %) [8]. Despite currently available advanced imaging technologies, it is difficult to diagnose gallbladder carcinomas at early stages due to their asymptomatic properties. Because gallbladder carcinomas are often diagnosed at advanced stages, curative resections are possible in only approximately 25 % of cases [10].

AFP is a serum glycoprotein that is frequently detected during pregnancy or in patients with HCC and germ cell tumors. AFP production has also been reported in malignant tumors of various organs originating from the foregut endoderm, such as the stomach, pancreas, lung, kidney, and urachus [11]. AFP is often used to detect recurrence or to evaluate treatment efficacy for AFP-producing tumors. According to one study, except for HCC and germ cell tumors, the stomach is the most common location of AFP-producing tumors, of which 43.5 % were poorly differentiated carcinomas and 6.5 % were signet ring cell carcinomas [12]. Considering that AFP is predominantly synthesized during embryogenesis, it is reasonable to expect that the ability to synthesize AFP would depend on the status of cellular differentiation, i.e., poorly differentiated cells and signet ring cells can contribute to high-level AFP secretion, while well-differentiated cells can only produce low levels of AFP [13].

Due to the similar developmental origin of the liver and gallbladder, gallbladder carcinoma could theoretically produce AFP in some cases. Only a few cases of AFP-producing gallbladder carcinomas have been reported in the literature, and most of these cases have exhibited papillary or hepatoid morphologies and poor prognosis [14]. Primary AFP-producing gallbladder carcinomas reported in the recent English literature consist of four histologic types: undifferentiated carcinoma, poorly differentiated carcinoma, clear cell carcinoma, and hepatoid carcinoma (Table 1) [15], [16], [17], [18]. In our patient, histopathological studies revealed poorly differentiated adenocarcinoma with signet ring cells, and immunohistochemical studies showed AFP production. Signet ring cell carcinoma is characterized by signet ring cells with intracytoplasmic mucin occupying >50 % of the tumor [19]. In our case, as the proportion of signet ring cells within the whole tumor was approximately 30 %, we diagnosed poorly differentiated adenocarcinoma with signet ring cells, and not signet ring cell carcinoma. Only a few cases of AFP-producing gallbladder carcinoma with signet ring cells have been reported. Immunohistochemical studies also yielded positive results for SALL 4 and Glypican 3, both of which are oncofetal proteins highly expressed in germ cell tumors. Although these proteins are often expressed in AFP-secreting gastric carcinoma, there are no reports of their expression in AFP-producing gallbladder carcinoma. Ushiku et al. suggested that SALL 4 positivity represents fetal gut differentiation rather than the existence of hepatocytes. SALL 4 is completely negative in HCC, and thus is especially useful for distinguishing AFP-producing tumors from HCC [20]. Our case is an example of an AFP-producing gallbladder carcinoma of poorly differentiated adenocarcinoma with signet ring cells as well as immunohistochemical AFP, SALL 4, and Glypican 3 positivity. Lower serum AFP levels were seen after cholecystectomy, while increased AFP levels were observed after transcatheter arterial chemoembolization and radiofrequency ablation for the liver tumors. Although we did not have the opportunity to histologically diagnose the liver tumors, the CE-CT scan at the time of HCC diagnosis showed no apparent tumor in the gallbladder, strongly suggesting tumors in the liver were not metastases from gallbladder carcinoma. Considering that no residual tumors in the liver or metastases were observed after surgery, we concluded that the elevated serum AFP levels were due to an AFP-producing gallbladder carcinoma.

**Table 1:** Recent reports of alpha-fetoprotein-producing gallbladder carcinomas.

Case no.	Publication	Age, sex	AFP (ng/mL)	Tumor size (mm)	Histology	Immunohistochemistry	Metastasis	Treatment	Follow up (month)
1	Dixit N et al. 2021 [18]	60, F	110	12 × 18	Clear cell carcinoma	AFP+, CK7+, CEA+, CK20+, PAX8-	None	Lap-Chol+ Chem	Alive (−)
2	Sentani K et al. 2014 [19]	78, F	125	45 × 35	Clear cell carcinoma	AFP+, synaptophysin-, chromogranin A-, NCAM-	None	Chol	Alive (8)
3	Karayiannakis AJ et al. 2012 [20]	60, F	62	6 × 5	Hepatoid carcinoma	AFP+, EMA+, pan-keratins+, CK8+, CK18+, CK19+	None	Chol (whole layer) + Chem	Death (10) due to recurrence
4	Fujii H et al. 2012 [21]	78, F	157,428	–	Undifferentiated adenocarcinoma	AFP+, CK7+, CK19+, synaptophysin+, chromogranin A+, CK20-, CK56-	Para-aortic lymph nodes	Chem	Death (2)
5	Shimada K et al. 2009 [22]	69, M	1495	90 × 50	Poorly differentiated adenocarcinoma	AFP+, cytokeratin+, vimentin+, α-SMA-, S-100-	None	Chol (whole layer)	Alive (6)
6	Kao CY et al. 2009 [16]	73, M	231	60 × 40	Hepatoid carcinoma	AFP+, CK7-, synaptophysin-, chromogranin A-	None	Chol	Death (21) due to upper gastrointestinal hemorrhage
7	Our case 2022	69, F	1474	20 × 20	Poorly differentiated adenocarcinoma with signet ring cells	AFP+, SALL 4+, Glypican 3+	None	Lap-Chol (whole layer)	Alive (4)

AFP: alpha-fetoprotein, CEA: carcinoembryonic antigen, α-SMA: α-smooth muscle actin, SALL 4, Sal-like protein 4, Lap: Laparoscopic, Chol: Cholecystectomy, Chem: chemotherapy.

AFP production would be associated with the status of cellular differentiation. Although AFP-producing gallbladder carcinoma is extremely rare, such tumors must be included in the differential diagnosis of patients who have elevated serum AFP level if no lesions are detected in other areas.

## 4 Conclusion

We report an extremely rare case of an AFP-producing gallbladder carcinoma of poorly differentiated adenocarcinoma with signet ring cells. The status of cellular differentiation would be associated with the ability to synthesize AFP. When no hepatic or other metastatic lesions can account for elevated AFP levels, AFP-producing gallbladder carcinoma should be included in differential diagnoses.

## Consent

Written informed consent was obtained from the patient for the publication of this case report and accompanying images. A copy of the written consent is available for review by the Editor-in-Chief of this journal on request.

## Funding

This research did not receive any specific grant from funding agencies in the public, commercial, or not-for-profit sectors.

## Ethical approval

Ethical approval has been exempted by our institution because this is a case report and no new studies or new techniques were carried out.

## Author contribution

JM: writing the manuscript.

KT: critical revision.

KF: final approval of the article.

All authors read and approved the final manuscript.

## Guarantor

Junya Mita.

## Declaration of competing interest

All the authors certify that there is no conflict of interest.
